# Prolonged Application of Continuous Passive Movement Improves the Postoperative Recovery of Tibial Head Fractures: A Prospective Randomized Controlled Study

**DOI:** 10.1155/2022/1236781

**Published:** 2022-02-16

**Authors:** Christiane Kabst, Xinggui Tian, Christian Kleber, Michael Amlang, Lisa Findeisen, Geoffrey Lee, Stefan Zwingenberger

**Affiliations:** ^1^University Center of Orthopaedic, Trauma and Plastic Surgery, University Hospital Carl Gustav Carus at Technische Universität Dresden, 01307 Dresden, Germany; ^2^Department of Orthopedics, Trauma Surgery and Plastic Surgery, University Hospital of Leipzig, 04103 Leipzig, Germany; ^3^Kennedy Institute of Rheumatology, Nuffield Department of Orthopedics, Rheumatology, and Musculoskeletal Sciences, University of Oxford, Oxford, UK

## Abstract

**Methods:**

60 patients with THFs were randomly and equally divided into the CPM group and non-CPM group. Both groups immediately received CPM and conventional physical therapies during hospitalization. After discharge, the non-CPM group was treated with conventional physical therapy alone, while the CPM group received conventional physical training in combination with CPM treatment. At 6 weeks and 6 months postoperatively, the primary outcome which was knee ROM and the secondary outcome which was knee functionality and quality of life were evaluated.

**Results:**

The CPM group had a significantly increased ROM at both follow-up time points. The Knee Society Score, UCLA activity score, and the EuroQoL as well as the pain analysis showed significantly better results of the CPM group than the non-CPM group.

**Conclusions:**

The prolonged application of CPM therapy is an effective method to improve the postoperative rehabilitation of THFs.

## 1. Introduction

Tibial head fractures (THFs) can be caused by high energy trauma incidents, mostly motor vehicle accidents, as well as low energy falls of geriatric patients with osteoporotic bone [1, 2]. They often require surgical treatment, and its main objectives are to restore the articular surface and axial relationships, avoid long-term immobilization, and ultimately restore the function of the injured knee joint as soon as possible [2, 3]. However, it is reported that the average motion of the knee is still limited 15 months after THF surgery, when compared with the healthy population (105° vs. 150°) [2, 4, 5]. Knee joint movement limitation caused by THFs severely restrict the patient's daily life, since high mobility is required to manage daily tasks, such as climbing stairs and sitting and standing from chairs requiring 90-120° of flexion and entering a bathtub requiring at least 135° of flexion [2, 6]. How to increase the range of motion (ROM) and the functionality of the knee as much as possible has become the focus of postoperative rehabilitation of THFs.

Continuous passive motion (CPM) is an external device that enables joints to move passively on a preset arc of motion [7]. Currently, CPM is widely used in postoperative rehabilitation that limits the ROM of joint, mainly including fracture repair [2, 8], rotator cuff repair [9], hand rehabilitation [10], reconstruction rehabilitation of the anterior cruciate ligament [11, 12], total knee arthroplasty (TKA) [7, 13], and adhesive capsulitis [14]. However, there is still no consensus on the clinical functional recovery outcome and standard intervention measures of CPM [7, 12, 15]. Regarding the rehabilitation of CPM for THFs, a study of intra-articular knee fractures involving proximal tibial fractures showed that, compared with the non-CPM group, the CPM group, which used CPM for 48 hours, had significantly increased knee ROM at short-term postoperation, but there was no significant difference at other longer follow-up time points [2]. However, whether prolonged CPM application affects postoperative rehabilitation after THFs remains unknown.

In addition, with the development of surgical technology and enhanced recovery after surgery, the patient's length of hospital stay has been reduced [16, 17], which led to a reduction of recovery time in the hospital. Rehabilitation at home becomes very important and a supplement to the insufficient time of rehabilitation in hospital. CPM is now more and more used in the postclinical home situation and has become a part of daily care plan [7]. The purpose of this study is to explore whether the prolonged application of CPM in the home situation will improve midterm postoperative rehabilitation after THFs. We hypothesized that prolonged use of CPM in the home situation is beneficial in the postoperative recovery of THFs.

## 2. Methods

### 2.1. Study Design

**Figure 1:**
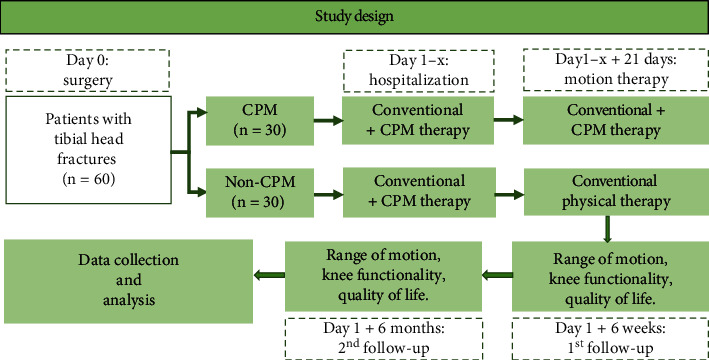
Schematic diagram of the processing method, timeline, and the evaluation parameters.

A prospective, nonblinded, controlled single-center trial of 60 patients who had been surgically treated for THF was performed at the University Hospital Carl Gustav Carus at TU Dresden, Germany. They were randomized into 2 groups of 30 patients each (CPM group and non-CPM group). CPM and conventional physical therapies started on the first postoperative day in both groups. Whereas the CPM group intensified its training with an additional CPM therapy for 21 days after discharge, the non-CPM group received conventional physical therapy only. Follow-up points were set 6 weeks and 6 months postoperatively (Figure 1).

### 2.2. Participants

This trial included all patients undergoing tibial head surgery from February 2017 to October 2018 at the University Hospital Carl Gustav Carus at TU Dresden, Germany. All patients had open reduction and internal fixation (ORIF), with the operative and fixation method decided by their surgeons. Further inclusion criteria were an age of 18 years or older, a radiologically assured THF (OTA 41 type A/B/C), free motion of the knee joint prior to injury. and a healthy, freely movable contralateral knee joint. All patients with a previous knee injury, pathological fracture, open tibial physis, pelvic fracture, spinal injury, hip injury, and other diseases hindering the use of CPM were excluded. Patients were randomly divided into the CPM group and non-CPM group on a one-to-one ratio by the block randomization method.

### 2.3. Interventions

All patients received conventional physical therapy and CPM therapies from the first postoperative day. The conventional physical therapy comprising of 30 minutes of training with stretching exercises and muscle strength (2-3 times/week) and CPM therapy were performed 3 times a day per 30 minutes. The CPM therapy was performed by using a Kinetec Optima S4 device (S&U Medizintechnik GmbH, Zottenheim, Germany). After being discharged from the hospital, the non-CPM group was treated with conventional physical therapy alone. In addition to the conventional physical training, the CPM group continued the same rehabilitation program during the hospitalization to enable home training for 21 days after being discharged from the hospital. The ROM of CPM could be set individually using a remote control. Altogether, a ROM from -10° to 120° was covered by the CPM device. All patients were encouraged to move their knee joints on the first day after the operation, with partial weight-bearing within the first six weeks and full weight-bearing thereafter.

### 2.4. Outcomes

The therapy was assessed at 6 weeks and 6 months postoperatively. The primary outcome of the investigation was the ROM of knee. For this purpose, a goniometer was used which measured the ROM of the injured and contralateral healthy knee. Knee functionality and the patient's quality of life were determined as secondary outcome measurements which were assessed by the Knee Society Score (KSS) [18], the Oxford Knee Score (OKS) [19], the EuroQoL [20], and the University of California at Los Angeles (UCLA) activity score difference. Specially, the UCLA activity score difference was the difference between the preinjury status score and follow-up time point score, and the scoring rules were based on the previous standards [21].

### 2.5. Sample Size

When calculating the sample size, ROM was used as the main outcome parameter. The average knee ROM after tibial head fracture was 105°, and the clinically significant increase of ROM was 15°. Using an alpha of 0.05, a statistical power of 80%, 20% rate of dropouts, and combined with previous studies [2], sample size of 60 patients had statistical significance and was used in this study.

### 2.6. Statistical Methods

The data were performed by using SPSS software (SPSS Inc., Chicago, IL, USA). The normality of distribution of continuous variables was tested by one-sample Kolmogorov-Smirnov test. Continuous variables with normal distribution were presented as mean ± standard deviation and range. The mean of ROM, knee flexion, knee extension, functional outcome score, and baseline data of the CPM group and non-CPM group were compared at each follow-up time point by independent samples Student's *t* test. The mean of ROM among 6 weeks and 6 months postoperative and the contralateral uninjured knee in the non-CPM group and CPM group were compared by a one-way ANOVA, followed by Tukey's post hoc test for multiple comparisons. A value of *p* < 0.05 was considered significant.

## 3. Results

### 3.1. Recruitment

From February 2017 to October 2018, 60 patients were recruited in this study. The follow-up ended 6 months postoperatively, because that is our typical follow-up time for every patient with THF. According to the internal hospital standards, follow-up of the patients was performed 6 months postoperatively. To assess short-term influence, a 6-week follow-up point was additionally chosen during which patients were just with partial weight-bearing. The trial was completed in April 2019. The baseline data for each group are represented in Table 1.

### 3.2. Participants

**Figure 2:**
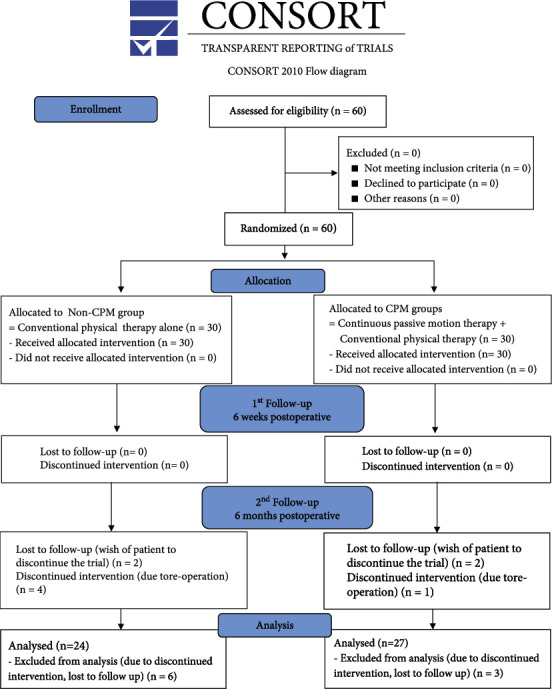
Consort (consolidated standards of reporting trials) flow diagram. Abbreviation: CPM: continuous passive motion.

60 patients were recorded and split into two treatment groups. All 60 patients completed 6-week follow-up. At 6 months postoperatively, group size of non-CPM group decreased from 30 to 24 due to follow-up loss. 27 out of the initial 30 patients were analyzed in the CPM group. Reasons were reoperation (non-CPM group: 4 patients and CPM group: 1 patient) and patients' request to discontinue the trial (non-CPM group: 2 patients and CPM group: 2 patients) (Figure 2).

### 3.3. Outcomes and Estimations

**Figure 3:**
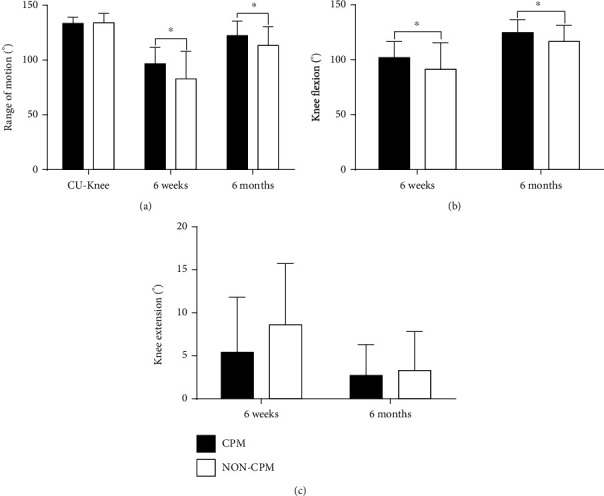
(a) Range of motion, (b) flexion, and (c) extension of the knee at 6 weeks and 6 months postoperatively and the range of motion of the uninjured knee. Abbreviations: CPM: continuous passive motion; CU-Knee: contralateral uninjured knee. Data are represented as mean ± standard deviation, ^∗^*p* < 0.05.

**Figure 4:**
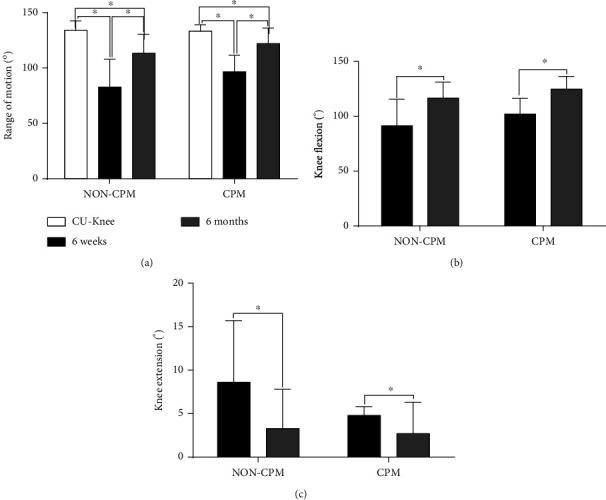
(a) Comparison of the range of motion among 6 weeks and 6 months postoperative and the contralateral uninjured knee in the non-CPM group and CPM group. Comparison the knee flexion (b) and knee extension (c) between 6 weeks and 6 months postoperative in the non-CPM group and CPM group. Abbreviations: CPM: continuous passive motion; CU-Knee: contralateral uninjured knee. Data are represented as mean ± standard deviation, ^∗^*p* < 0.05.

**Figure 5:**
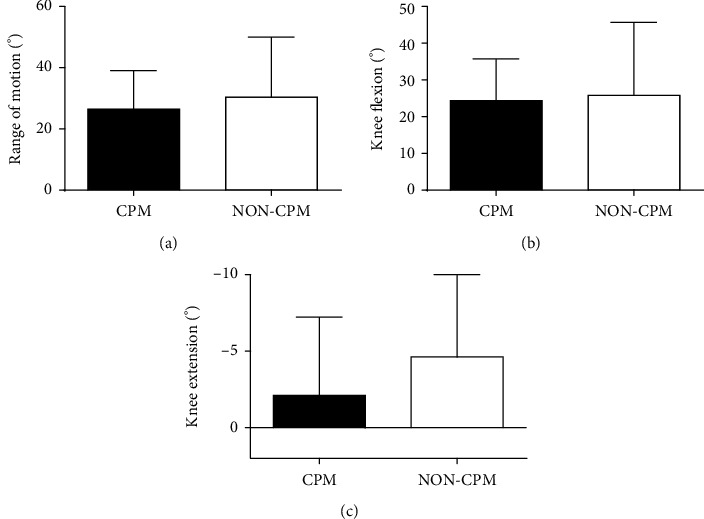
Comparison of the improvements from 6 weeks to 6 months (a, range of motion; b, knee flexion; c, knee extension) between non-CPM and CPM groups.

The ROM of the contralateral uninjured knee in the CPM group (133.4 ± 5.6) and the non-CPM group (134.1 ± 8.4) was equivalent (*p* = 0.583). At 6 weeks after surgery, the CPM group had a significant increase in ROM compared with the non-CPM group (CPM group vs. non-CPM group; 96.7 ± 14.8° vs. 82.8 ± 25.1°, *p* = 0.012). The different values could also be observed for knee flexion (non-CPM group vs. CPM group; 91.4 ± 24.1° vs. 102.0 ± 14.5°, *p* = 0.042) and knee extension (non-CPM group vs. CPM group; 8.6 ± 7.1° vs. 5.4 ± 6.4°, *p* = 0.073). At 6 months after surgery, the CPM group also had a significant increase in ROM compared with the non-CPM group (CPM group vs. non-CPM group; 122.4 ± 13.2° vs. 113.4 ± 17.1°, *p* = 0.040). The knee flexion of the non-CPM group appeared to be significantly smaller than that of the CPM group (non-CPM group vs. CPM group; 116.7 ± 14.6° vs. 124.8 ± 11.6°; *p* = 0.032). The extension of CPM patients (2.7 ± 3.6°) was only marginally better than that of the non-CPM group (3.3 ± 4.5°) (*p* = 0.633) (Figure 3). In addition, both in the non-CPM and CPM groups, the recovery effect of 6 months after surgery was better than that of 6 weeks after surgery, including ROM, flexion, and extension of the knee. However, the ROM of injured knee joint in the two groups was still lower than the ROM of contralateral knee joint even after 6 months (Figure 4). Comparing the improvements from 6 weeks to 6 months after surgery, it was found that the motion of the knee, including ROM, flexion, and extension, in the CPM group was not better than that of in the non-CPM group (Figure 5). There were significant differences in the analysis of KSS and the EQ-5D-3L part of EuroQoL score at both follow-up time points. OKS and visual analog scale (VAS) part of the EuroQoL score in the CPM group showed better results than the non-CPM group, but there was only a significant difference at 6-month follow-up. At both follow-up time points, the pain score of the CPM group also showed better results in pain points of KSS. The UCLA activity score difference demonstrated better results in the CPM group, but there was no significant difference at both time points (Table 2). The improvements of knee functionality and the patient's quality of life from 6 weeks to 6 months postoperation showed that the results were similar between the two groups, and there was no statistical difference in all results (Table 3).

### 3.4. Harms

No direct harms and unintended effects due to physical therapy occurred in both groups. In total, 5 patients underwent reoperations for the following reasons: repeated trauma (non-CPM group: 1 patient), revision of osteochondral defect (non-CPM group: 1 patient), and revision by compartment syndrome (CPM-group: 2 patients and non-CPM group: 1 patient).

## 4. Discussion

Compared with the non-CPM group, the results of this study showed that the CPM group had a significant increase in ROM, enhanced knee functionality, lower pain, and improved quality of life at the two follow-up time points. Thus, the hypothesis that prolonged application of CPM in the home situation in the postoperative treatment of THFs is beneficial was verified.

THFs are a type of common and severe injury, and their later developmental complications such as traumatic arthritis, muscle and bone atrophy, and joint stiffness can cause functional problems in the knee of the patients and increase the socioeconomic burden [22]. A systematic review demonstrated that tibial plateau fractures have a lower return rates to sport compared with other types of fractures, and only 60% patients can recover to the preinjury level of sport [23]. Another study found that 88% of patients suffering from tibial plateau fractures involving the posterior column cannot recover to their preinjury levels of activity, and their restricting factors include pain (66%), fear of reinjury (37%), limited ROM (26%), and instability (21%) [24]. Therefore, the promotion of THF rehabilitation has important significance, and the attention should be paid to not only ROM but also other restricting factors such as pain and quality of life in the process of rehabilitation of THFs.

Some preclinical studies demonstrated that CPM can prevent joint stiffness, and its underlying mechanics is that it can produce sinusoidal intra-articular pressure changes that promote trans synovial transport and clearance of the blood to prevent edema formation, halting granulation, and fibrotic tissue formation [15, 25]. In addition, CPM has the potential to limit muscle atrophy and relieve pain [15, 26, 27]. In clinical work, the early mobilization and increased ROM of the knee after THF surgery is important [22], and CPM is often used for postoperative rehabilitation of patients with tibial fractures after surgical fixation [28]. Surprisingly, there is only one report on this study between the CPM and THFs which showed that CPM therapy significantly increased the knee motion after short-term use of CPM, and its effect on enhanced rehabilitation after THF also immediately ended when CPM application was discontinued [2]. Another study showed that 3 days of CPM use could influence joint stiffness up to 24 weeks [29], so prolonged use of the CPM appears to be meaningful for post-THF rehabilitation. In this study, motion therapy started on the first postoperative day for both groups which can reduce the swelling and stiffness of the joints. However, there has been no consensus about the usage, duration, and timing of CPM therapy in all areas of application yet [30, 31]. A medium application duration of three times a day for 30 minutes during hospital and the continuing first 3 weeks after being discharged from the hospital was chosen, and this also enabled patients to have good compliance. A Cochrane review concluded that using CPM and physiotherapy has more beneficial short-term results than physiotherapy alone after TKA [32]. In our study, the CPM combination with conventional physical training was used for the recovery of THFs to hope for a good recovery for the patients.At 6 weeks and 6 months after surgery, the CPM group had a significant increase in ROM, extension, and flexion compared with the non-CPM group. This may be related to the prevention of edema, granulation tissue, and fibrotic tissue formation by CPM [15, 25]. In addition, it also may be related to CPM improving tendon strength, cartilage repair, and wound healing [33–35]. Apart from this, this study showed that CPM significantly decreased the patients' level of pain which was consistent to other findings [11, 12]. The reasons might be a decreased inflammation and mitigated hyperalgesia [36–39]. The improvement in the CPM group from 6 weeks to 6 months after the operation was slightly lower than that of in the non-CPM group without a significant difference. One reason for the slightly slowed improvement may be due to the improved knee mobility in the early stage resulting in a lower increase in the later period in the CPM group. Another fact that cannot be ignored is that after 6 weeks postsurgery, all patients transitioned from partial weight-bearing to full weight-bearing exercise. A review study showed that weight-bearing after knee surgery can appropriately stimulate knee healing, reduce pain, and improve activity level [40]. However, the specific impact of weight-bearing on knee mobility, knee function, and quality of life after knee surgery needs further research. The EuroQoL could provide mobility, self-care, main activity, social relationships, pain, and mood values [41]. The OKS and KSS for knee functionality suggested by the German Orthopedic Guidelines (Deutsche Gesellschaft für Orthopädie und Orthopädische Chirurgie 2018) and the UCLA activity rating are valid for clinical activity assessments [21]. All these findings have a positive impact on the patients' quality of life and knee functionality and were positively influenced by the prolonged CPM treatment. The fact that there was no significant difference between the CPM group and the non-CPM group in the development from 6 weeks to 6 months postoperative in quality of life and knee functionality shows that the positive effect of the additional CPM therapy in the first 3 weeks at home remains an advantage even after 6 months. The observed differences in knee flexion can also been seen as clinically relevant since a patient with 90° of knee flexion has difficulties to go down on his knees while 10° more flexion make that much easier.

Moreover, some studies could observe that hemarthrosis and deep vein thrombosis occurred less [42] and the number of manipulations under anesthesia were decreased [31] using CPM. In this study and another intra-articular fractures recovery study [2], the incidence of postoperative manipulations under anesthesia and thrombosis was rare regardless of whether CPM was used. Therefore, this study cannot draw similar conclusion, and it requires further research with large samples and multicenter trial.

## 5. Limitations

In this study, there were considerably more women than men in the CPM group, and it cannot preclude influence on the data. The last follow-up time point was set after 6 months postoperatively giving insufficient information about the long-term benefit of CPM therapy. This study was a single-center study with a small sample size, and a large sample multicenter randomized trial would be useful to further confirm our results.

## 6. Conclusions

The prolonged application of CPM therapy in combination with conventional physical therapy at home in treatment of THFs increased the ROM of the knee, reduced pain, and improved the knee functionality and quality of life of patients. In conclusion, the prolonged application of CPM therapy in the home situation is an effective method to promote postoperative rehabilitation of THFs.

## Figures and Tables

**Table 1:** Baseline data of the CPM and non-CPM group.

	CPM	Non-CPM	*p*
Age (years)	56.6 ± 16.1	56.0 ± 13.8	0.877
	(27–86)	(27–81)	
Gender	10 males	15 males	
	20 females	15 females	
Body weight (kg)	75.1 ± 11.8	81.4 ± 16.5	0.095
	(55–106)	(57–118)	
Operation time (minutes)	147.3 ± 55.2	140.6 ± 71.1	0.683
ASA classification	1.77 ± 0.6812	1.73 ± 0.64.12	0.846
	(1–3)	(1–3)	
OTA classification of the fracture	B2: 3	A1: 1	
	B3: 12	B1: 3	
	C3: 15	B2: 2	
		B3: 11	
		C2: 2	
		C3: 11	
Time in hospital (days)	13.9 ± 1.2	15.4 ± 1.6	0.5
	(5–31)	(7–49)	

Abbreviations: CPM: continuous passive motion; ASA: American Society of Anesthesiologists; OTA: Orthopedic Trauma Association. Data are represented as mean ± standard deviation and range; significance was set at *p* < 0.05.

**Table 2:** Overview of the Knee Society Score, EuroQoL, Oxford Knee Score, and UCLA activity score difference results.

	6 weeks			6 months		
	CPM	Non-CPM	*p*	CPM	Non-CPM	*p*
KSS^1^	125.8 ± 33.3	105.8 ± 38.0	0.034	179.5 ± 29.3	151.1 ± 28.6	0.001
Pain points of KSS^1^	38.8 ± 11.0	30.7 ± 12.1	0.008	44.8 ± 7.0	36.7 ± 12.1	0.004
EQ-5D-3L of EuroQoL score	7.4 ± 1.6	8.6 ± 1.7	0.005	6.1 ± 1.4	7.1 ± 1.4	0.013
VAS^2^ of EuroQoL score	66.2 ± 17.0	58.2 ± 19.0	0.092	83.9 ± 11.4	75.8 ± 11.0	0.014
UCLA^3^ activity score difference	3.2 ± 1.1	3.3 ± 1.4	0.686	1.0 ± 1.0	1.4 ± 1.1	0.190
OKS^4^	28.0 ± 9.1	24.9 ± 9.8	0.207	39.3 ± 6.3	33.2 ± 7.1	0.002

Abbreviations: CPM: continuous passive motion; KSS^1^: Knee Society Score; VAS^2^: visual analogue scale; UCLA^3^: University of California at Los Angeles; OKS^4^: Oxford Knee Score. Data are represented as mean ± standard deviation; significance was set at *p* < 0.05.

**Table 3:** Comparison of the improvements from 6 weeks to 6 months between the non-CPM and CPM groups.

	CPM	Non-CPM	*p*
KSS^1^	51.1 ± 18.6	44.3 ± 31.5	0.345
Pain points of KSS^1^	5.2 ± 8.5	5.8 ± 13.6	0.837
EQ-5D-3L of EuroQoL score	−1.2 ± 1.6	−1.5 ± 1.7	0.501
VAS^2^ of EuroQoL score	17.0 ± 15.5	14.1 ± 13.1	0.476
UCLA^3^ activity score	2.1 ± 1.0	1.7 ± 1.0	0.109
OKS^4^	11.0 ± 8.2	7.5 ± 8.8	0.142

Abbreviations: CPM: continuous passive motion; KSS^1^: Knee Society Score; VAS^2^: visual analogue scale; UCLA^3^: University of California at Los Angeles; OKS^4^: Oxford Knee Score. Data are represented as mean ± standard deviation; significance was set at *p* < 0.05.

## Data Availability

All data generated or analyzed during this study are included in this article.

## Abbreviations

THF: Tibial head fracture
CPM: Continuous passive motion
ROM: Range of motion
TKA: Total knee arthroplasty
ORIF: Open reduction and internal fixation
KSS: Knee Society Score
OKS: Oxford Knee Score
UCLA: University of California at Los Angeles
ASA: American Society of Anesthesiologists
OTA: Orthopedic Trauma Association.
